# DEPRESSIVE TENDENCIES AND NEGATIVE LIFE EVENTS AS PREDICTORS OF HOMEBOUND STATUS AMONG OLDER ADULTS IN JAPAN

**DOI:** 10.1093/geroni/igad104.2687

**Published:** 2023-12-21

**Authors:** Koji Fujita, Sachiko Yamazaki, Hiromi Imuta, Roseline Yong, Hiroko Matsunaga, Yoshinori Fujiwara

**Affiliations:** Tokyo Metropolitan Institute for Geriatrics and Gerontology, Tokyo, Tokyo, Japan; Bunkyo Gakuin University, Fujimino, Saitama, Japan; Tokyo Metropolitan University, Arakawa, Tokyo, Japan; Akita University, Akita, Akita, Japan; Tokyo Metropolitan Institute of Gerontology, Tokyo, Tokyo, Japan; Tokyo Metropolitan Institute for Geriatrics and Gerontology, Tokyo, Tokyo, Japan

## Abstract

This study aimed to examine negative life events (NLEs) and depressive tendencies as predictors of homebound status, which poses a risk of long-term frailty in older adults. A population-based cohort study (2018-2020) using self-administered questionnaires was conducted with older adults (65-94 years) in the rural community of Akita Prefecture, Japan. The response rate at the baseline survey in 2018(T1) was 61.5%. About 81.1% (1,048 out of 1,291) of the participants at T1 responded to the follow-up survey in 2020 (T2). Among them, 77.3% (810) were not homebound at T1. After data cleaning, 790 participants’ data were analyzed. A frequency of going out less than once a week was considered a homebound status. In this study, NLEs included 1) loss of a close relative, 2) major illness or injury to oneself, 3) major illness or injury to a family member, 4) financial difficulties, and 5) loss of one’s role (family, workplace, society, others). Depressive tendencies were evaluated using the 6-item Kessler Psychological Distress Scale (0-24 scale). A K6 score ≥ 9 indicates depressive tendencies. Logistic regression analysis was adjusted for potential confounders. The incidence of being homebound at two years was 10.1% for men and 17.1% for women, with a significant gender difference (p< 0.01). The adjusted odds ratio for depressive tendencies was 3.37 (95%CI= 1.62-7.01) among homebound (vs. non-homebound) individuals. However, the five NLEs were not statistically significant. Our results suggest that depressive tendencies, but not NLEs, can cause older adults to remain housebound.

